# Study protocol of a multicenter phase III randomized controlled trial investigating the efficiency of the combination of neoadjuvant chemotherapy (NAC) and neoadjuvant laparoscopic intraperitoneal hyperthermic chemotherapy (NLHIPEC) followed by R0 gastrectomy with intraoperative HIPEC for advanced gastric cancer (AGC): dragon II trial

**DOI:** 10.1186/s12885-020-6701-2

**Published:** 2020-03-17

**Authors:** Maneesh Kumarsing Beeharry, Zhen-Tian Ni, Zhong Yin Yang, Ya Nan Zheng, Run Hua Feng, Wen-Tao Liu, Chao Yan, Xue Xin Yao, Chen Li, Min Yan, Zheng-Gang Zhu

**Affiliations:** Shanghai Key Laboratory of Gastric Neoplasms, Shanghai Institute of Digestive Surgery, Department of Surgery, Rui Jin Hospital, Shanghai Jiao Tong University School of Medicine, Shanghai, 200025 China

**Keywords:** Advanced gastric Cancer, Intraperitoneal Hyperthermic chemotherapy, Neoadjuvant chemotherapy, Peritoneal Carcinomatosis, Progression-free survival

## Abstract

**Background:**

Even though treatment modalities such as adjuvant systemic radio-chemotherapy and neoadjuvant chemotherapy (NAC) have individually have improved overall survival (OS) and progression-free survival (PFS) rates in advanced Gastric Cancer (AGC), the peritoneum still presides as a common site of treatment failure and disease recurrence. The role of hyperthermic intraperitoneal chemotherapy (HIPEC) has been acknowledged as prophylaxis for peritoneal carcinomatosis (PC) in AGC patients and in this study, we aim at investigating the safety and efficacy of the combination of neoadjuvant laparoscopic HIPEC (NLHIPEC) with NAC in the neoadjuvant phase followed by surgery of curative intent with intraoperative HIPEC followed by adjuvant chemotherapy (AC).

**Methods:**

In this multicenter Phase III randomized controlled trial, 326 patients will be randomly separated into 2 groups into a 1:1 ratio after laparoscopic exploration. The experiment arm will receive the proposed comprehensive Dragon II regimen while the control group will undergo standard R0 D2 followed by 8 cycles of AC with oxaliplatin with S-1 (SOX) regimen. The Dragon II regimen comprises of 1 cycle of NLHIPEC for 60mins at 43 ± 0.5 °C with 80 mg/m^2^ of Paclitaxel followed by 3 cycles of NAC with SOX regimen and after assessment, standard R0 D2 gastrectomy with intraoperative HIPEC followed by 5 cycles of SOX regimen chemotherapy. The end-points for the study are 5 year PFS, 5 year OS, peritoneal metastasis rate (PMR) and morbidity rate.

**Discussion:**

This study is one of the first to combine NLHIPEC with NAC in the preoperative phase which is speculated to provide local management of occult peritoneal carcinomatosis or peritoneal free cancer cells while NAC will promote tumor downsizing and down-staging. The addition of the intraoperative HIPEC is speculated to manage dissemination due to surgical trauma. Where the roles of intraoperative HIPEC and NAC have individually been investigated, this study provides innovative insight on a more comprehensive approach to management of AGC at high risk of peritoneal recurrence. It is expected that the combination of NLHIPEC with NAC and HIPEC will increase PFS by 15% and decrease PMR after gastrectomy of curative intent.

**Trial registration:**

World Health Organization Clinical Trials - International Registry Platform (WHO-ICTRP) with Registration ID ChiCTR1900024552, Registered Prospectively on the 16th July, 2019.

## Background

Gastric cancer (GC) is the fourth most common malignancy worldwide [1] but almost half of GC-related deaths in the world occur in China [2]. However, despite the triggering advances in medical research and technology, the prognosis of advanced GC (AGC) remains poor. Neoadjuvant chemotherapy (NAC) has been suggested for resectable AGC based on well-known Randomized Controlled Trial (RCT) s [3, 4]. Systemic adjuvant radio-chemotherapy following surgery increased overall survival (OS) rate by 32% and progression-free survival (PFS) rate by 51% [5] while the addition of perioperative chemotherapy to surgery decreased mortality by 25% and disease progression by 34% [3]. Nevertheless, the peritoneum still presides as a common site of treatment failure for AGC and at time of death, 60% of GC patients will have peritoneal dissemination: linked to significant quality of life impairment from complications such as tense ascites, malignant bowel obstruction, malnutrition and cachexia [6, 7]. Chemotherapy and immunotherapy alone have limited efficacy against peritoneal carcinomatosis. Thus, multimodality strategies including various combinations of systemic chemotherapy and hyperthermic intraperitoneal chemotherapy (HIPEC) have been studied to improve survival and prevent morbid complications.

In order to appreciate the prophylactic role of HIPEC, it is important to understand the pathogenesis of PC following surgery of curative intent in AGC: Intra-abdominal recurrence after curative resection usually originates from intraperitoneal free cancer cells, which in turn can occur from the spontaneous exfoliation of cancer cells from the primary tumor, or the traumatic dissemination of cancer cells as a result of the surgical trauma [8–10]. Sugarbaker et al. proposed the “tumor cell entrapment hypothesis” [9] according to which the intraperitoneal free cancer cells (IFCC) adhere to the surgical raw area within minutes by fibrin entrapment and assisted by cytokines released as part of the wound healing mechanism and the hypoxic environment renders the trapped cancer cells relatively immune to the effects of systemic chemotherapy. Hence the advent of regional intraperitoneal chemotherapy is therefore intended to clear these free cancer cells which persist after a curative resection [10]. While the large volumes of fluid used during HIPEC dilutes the intraperitoneal free cancer cells, the intraperitoneal administration of chemotherapy results in a positive gradient of chemotherapy in the peritoneum [11]. The hyperthermia synergistically enhances the effects of intraperitoneal chemotherapy by both direct cytotoxic action (impaired DNA repair, denaturation of proteins and increase in the lysosomal activity within the tumor cells) and indirect cytotoxic effects (increased penetration of the drug into the tumor nodule and increased drug uptake in the tumor cells), the cytotoxic activity of perioperative intraperitoneal chemotherapy destroys the cancer cells within the fibrin produced as part of the wound healing process [12].

The use of HIPEC to prevent peritoneal recurrence was first reported in 1988 by Koga et al. where a significant improvement in the 3-year survival (74% vs 53%, *p* < 0.04) and improvement in the peritoneal recurrence rates (36% vs 50%) was observed in patients who received prophylactic HIPEC after a curative gastrectomy [13]. These findings triggered more curiosity about the application of such a multimodality treatment option worldwide. During the period 1992–2002, 128 GC patients with peritoneal dissemination underwent surgery in our hospital were included in an HIPEC experiment and the 5-year survival rates were 5.5% for patients in the resection group and 0% for patients in the non-resection group (*P* < 0.001) [14]. In another randomized controlled trial from our faculty, the 1, 2 and 4 years survival rates with prophylactic HIPEC 85.7, 81.0 and 63.9% versus for surgery alone: 77.3, 61.0 and 50.8%. The peritoneal recurrence was control vs. HIPEC group 34.7% vs. 10.3% [15]. Over the last few years, there have been several high-quality meta-analyses weighing the role of HIPEC as a prophylaxis and cure to secondary PC, and there was consistency in acknowledging that HIPEC could effectively improve the survival rates of patients without peritoneal carcinomatosis (PC) while its role in patients with PC was rather limited [16–18].

With the potential benefits of primary tumor down-staging and lymph node metastasis and occult micro-metastases control in GC patients with better tolerance in the pre-operative stages, the concept of NAC promised better understanding and control on the biological behavior of tumor progression and therapeutic response [19]. The Intergroup 0116 study was the first to show the significant overall survival benefits of adjuvant chemo radiation therapy for GC [20] and the next study was the MAGIC trial which evaluated the efficacy of perioperative adjuvant chemotherapy [3]. Although the findings from the Intergroup 0116 and the MAGIC trial were positive, following studies such as ARTIST and EORTC 40954 studies found no significant survival benefits for AGC but EORTC 40954 demonstrated an increase in the radical resection rate in favor of T3-4N + M0 AGC undergoing NAC [4, 19]. In the FNCLCC/FFCD phase III trial, the 5-year survival rates were 24% in the surgery-alone arm and 38% in the perioperative chemotherapy arm (*p* = 0.02) [21]. In 2013, a Cochrane single patient data meta-analysis including 14 randomized trials showed an improvement in survival (HR = 0.81, 95%CI: 0.79–0.89, *P* < 0.0001) with a 5-year survival gain of 9% with a 1.4 times radical resection rate favoring the NAC arm [22]. Recently, the German FLOT4 trial established the perioperative FLOT regimen increased rates of curative surgery and prolonged median PFS and median OS as compared to the ECF/ECX (epirubicin/cisplatin/oral capecitabine) regimen [22, 23].

With HIPEC gaining more recognition as a prophylaxis against PC following surgery of curative intent in AGC patients, there were more speculations about the development of more comprehensive approaches allowing broader and more precise clinical management of AGC. In a study by Cui et al., 192 AGC patients were randomly divided into the following four groups (*n* = 48 per group): Control, NAC, HIPEC and joint groups and the results indicated that NAC combined with HIPEC for the treatment of AGC is well tolerated and exhibits improved compliance and efficiency [24]. While the efficacy of perioperative chemotherapy has been largely investigated and recognized [3–6], the concept of neo-adjuvant HIPEC as a prophylaxis against PC in the clinical management of AGC is yet recent. Henceforth, in this study, we aim to investigate the efficacy of the combination of neo-adjuvant laparoscopic HIPEC with NAC followed by surgery of curative intent with intraoperative HIPEC in AGC patients with serosal involvement with/out occult peritoneal dissemination. While the neoadjuvant L-HIPEC acts as a prophylaxis against occult peritoneal dissemination, the NAC promotes tumor down-staging and downsizing and the intra-operative HIPEC acts as a prophylaxis against peritoneal dissemination due to surgical trauma. In this study, we chose the control to be surgery only since despite the evidences supporting the efficacy of NAC in the management of AGC, NAC has not yet been widely recommended in the Asian guidelines.

## Methods/design

Dragon II is a multicenter randomized controlled trial which will be carried out in multiple hospitals throughout China. Eligible patients with locally advanced GC will be randomized into 2 groups to undergo D2 surgery with curative intent followed by adjuvant chemotherapy or undergo L-HIPEC+NAC followed by D2 surgery of curative intent with intraoperative prophylactic HIPEC and adjuvant chemotherapy. The study has been approved by the Ethics committee of Ruijin Hospital affiliated to Shanghai Jiao Tong University School of Medicine has already been registered in the World Health Organization Clinical Trials - International Registry Platform (WHO-ICTRP) with Registration ID ChiCTR1900024552. All patients entering the study would be required to sign informed consent. Monitoring will be carried out throughout the trial.

**Fig. 1:**
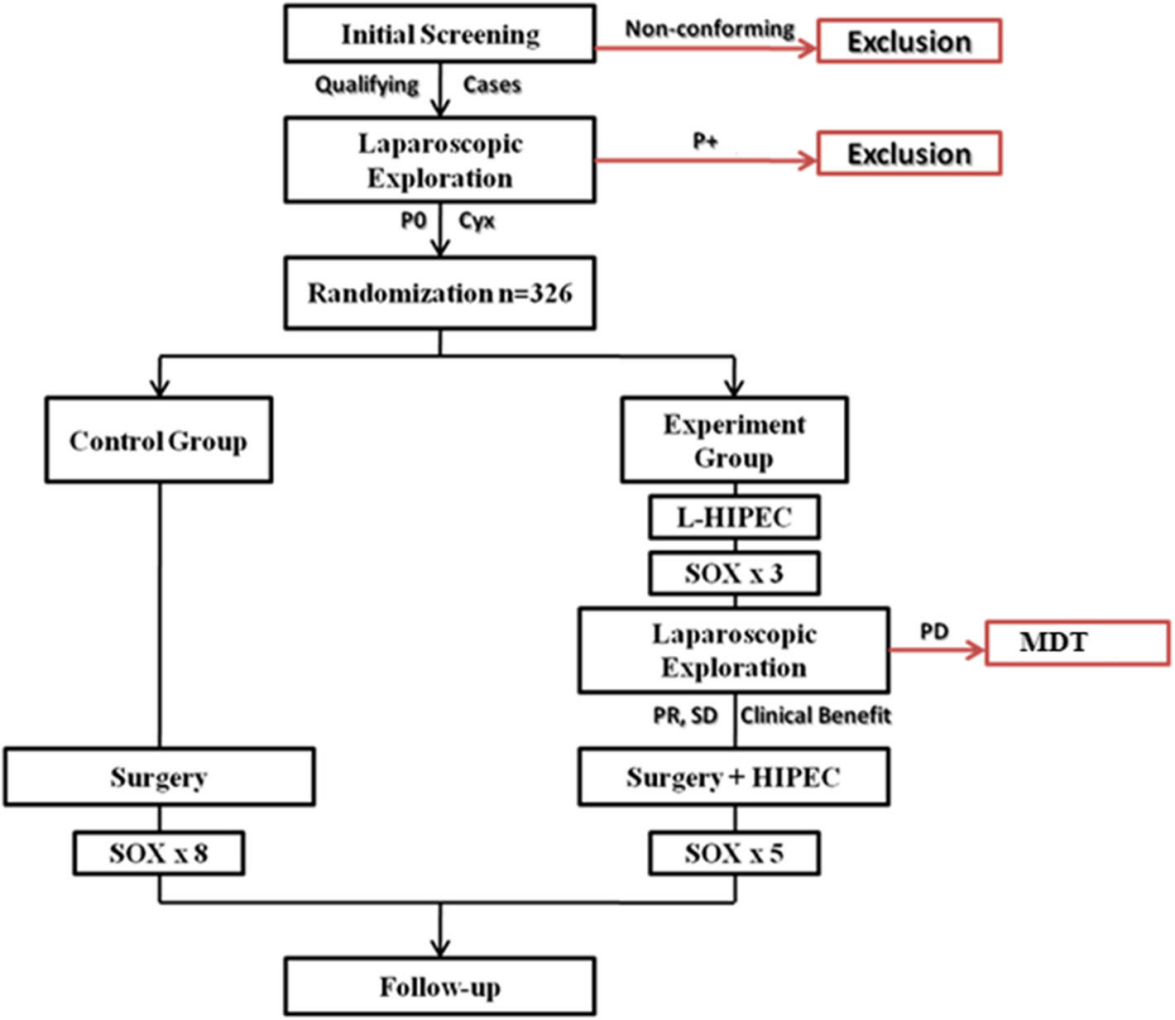
Study Flow Chart

**Protocol Overview** (Fig. 1)

**Primary endpoint**: Progression-free survival (PFS)

**Secondary endpoint**s: Overall Survival (OS), Peritoneal Metastasis Rate, Gastrectomy Radicality Rate, Post-operative Complications.

### Patient eligibility

#### Inclusion criteria

1. Aged between 18 and 75 years old;
2. Eastern Cooperative Oncology Group (ECOG) score ≤ 2;
3. American Society of Anesthesiologists (ASA) grades I-III;
4. Primary GC without prior history of gastric malignancy;
5. Multiple detector computed tomography (MDCT), Endoscopic Ultrasonography or Laparoscopic Exploration revealing lesion(s) infiltrating the serosal (T staging T4);
6. Normal Bone Marrow, Liver, Renal functions conforming to the following standards:
  - Peripheral blood white blood cells (WBC) count ≥3500/mm^3^, platelet count (PLT) ≥100,000/mm^3^ and hemoglobin count (Hb) ≥ 90 g/L;
  - Total bilirubin ≤1.5 times the upper limit of normal (ULN); Aspartate aminotransferase (AST) and alanine aminotransferase (ALT) ≤2.5 × ULN; Serum creatinine (SCr) ≤50 ml/min.
7. Negative urine or blood pregnancy test for female subjects of childbearing potential;
8. Expected life expectancy ≥3 months;
9. Willing to sign inform consent for participation and publication of results.

#### Exclusion criteria

1. Confirmed of evidence of distant metastasis (e.g. liver metastasis, lung metastasis, para-aortic lymph node metastasis, etc.)
2. Evidence of Peritoneal metastasis, ovarian metastasis or malignant ascites during laparoscopic examination;
3. Recurrent GC;
4. Primary gastric cancer has already been resected;
5. Pregnant or lactating women; Subjects of childbearing potential refusing to take contraceptive measures;
6. Serious uncontrolled recurrent infections or HIV infections.
7. Replicators of hepatitis B or C virus.
8. Clinically severe (i.e. active) heart disease with myocardial infarction or unstable angina pectoris within 6 months.
9. History of uncontrolled diabetes;
10. History of other malignant diseases in the last 5 years;
11. Clinically severe (i.e. active) heart disease, such as symptomatic coronary heart disease, New York Heart Association (NYHA) class II or more severe congestive heart failure or arrhythmia requiring drug intervention, or a history of myocardial infarction in the last 12 months.
12. Upper gastrointestinal obstruction or abnormal physiological function or mal-absorption syndrome may affect S-1 absorbers;
13. Known peripheral neuropathy (> NCI-CTC AE 1). However, patients with only disappearance of deep tendon reflex (DTR) need not be excluded;
14. Patients on steroid treatment after organ transplant;
15. Moderate or severe renal damage [SCr ≥ 50 ml/min], or SCR > ULN);
16. Anaphylaxis to Paclitaxel, oxaliplatin, tegiol or any research drug ingredient;
17. Patients unwilling to sign informed consent to participate and publish results.

## Treatments

### Laparoscopic exploration

Diagnostic laparoscopy was performed to define a baseline status of the extent of Peritoneal Carcinomatosis (PC) prior to enrolment according to standard requirements [25] where the primary lesion, the peritoneal cavity, liver, diaphragm, serosal surfaces, peritoneum, omentum, and pelvic organs are systematically inspected with lavage cytology examination. In case of no ascites, peritoneal lavage is performed using 300 ml of normal saline instilled into the right and left upper quadrants and pelvis; and washings ware collected for cytology examination.

#### Randomization and grouping

After the laparoscopic exploration confirming serosal involvement without peritoneal carcinomatosis, randomization is performed. Randomization is carried out by computer generated allocation in a 1:1 design protocol. According to study design, patients are separated into 2 arms:

Arm A: The Control Group where the patient undergoes standard R0 Gastrectomy followed by 8 cycles of adjuvant chemotherapy (AC) after recovery.

Arm B: The patient first undergoes L-HIPEC followed by 3 cycles of NAC. After clinical assessment and second diagnostic exploration rule out disease progression, subject undergoes standard R0 Gastrectomy plus intraoperative HIPEC. After recovery, subjects receive 5 more cycles of AC.

#### Surgery

A standard R0 open gastrectomy is recommended. The type of gastrectomy performed depends on the location and extent of the primary lesion according to recommendations by previous lterature [6]. For patients from the experiment group, the second look laparoscopic exploration will be scheduled at around 3 weeks after the last dosage of S-1.

#### L-HIPEC

The equipment for the laparoscopic and intraoperative procedures is the BR-TRG-I Hyperthermic Perfusion Intraperitoneal Treatment system (Baorui Medical Technology, Co., Ltd., Guangzhou, China). The laparoscopic HIPEC technique consists in through 15 mm trocars placing two inflow catheters in the upper abdominal region and two outflow catheters in the Douglas pouch, each catheter being connected to the corresponding thermo-probe. Once the randomization is performed and verified, the L-HIPEC procedure is started, a 43 ± 0.5 °C solution of Paclitaxel 80 mg/m^2^ being introduced into the peritoneal cavity and re-circulated for 60 min at a flow rate controlled between 400 and 600 ml. The chemotherapeutic agent is introduced on the two inflow catheters which are placed in the upper abdominal quadrant, in the proximity of the tumor bed and aspirated on the two outflow catheters which were placed in the Douglas pouch. The intra-abdominal temperature is monitored during the procedure by the four thermocouples which are placed at the level of the inflow and the outflow catheters respectively and is maintained at 43 ± 0.5 °C. In the meantime, the endo-esophageal temperature level is closely monitored (a value higher than 39 °C enforcing ending the procedure). After the procedure, the 4 catheters are removed and the trocars incisions closed.

#### Neoadjuvant chemotherapy

The NAC regimen is SOX, consisting of S-1 and oxaliplatin. Oxaliplatin 130 mg/m^2^ is administered intravenously on day 1. S-1 is administered orally with 40–60 mg, twice a day for 14 consecutive days, followed by a 7-day rest period. The dose of S-1 was accorded to body-surface area (BSA): patients with a BSA of less than 1.25 m^2^ receive 80 mg daily; those with a BSA of 1.25 m^2^ or more but less than 1.5 m^2^ receive 100 mg daily; and those with a BSA of 1.5 m^2^ or more receive 120 mg daily.

#### Adjuvant chemotherapy

The AC regimen is also SOX, implemented as described in the NAC section. The status and recovery of the patient would be assessed at a time-point set at around 3 weeks post-op and depending on the status of the patient; the post-op course of treatment will be scheduled.

#### Intraoperative HIPEC

After the anastomosis, the open coliseum technique is used, using Paclitaxel at a dose of 80 mg/m^2^ dissolved in 3–5 L of normal saline heated to 43 ± 0.5 °C, and infused into the abdominal cavity and re-circulated for 60 min at a flow rate controlled between 400 and 600 ml. After the HIPEC procedure, the abdomen is closed.

### Tumor response and toxicity criteria

In the Experiment arm, tumor response evaluations are taken after the third cycle of preoperative SOX by using abdominal MDCT scan. All these evaluations are done according to the Response Evaluation Criteria for Solid Tumors (RECIST) 1.1. Adverse events were assessed according to Common Terminology Criteria for Adverse Event (CTCAE) v4.0. In case of disease progression after the 3 cycles of NAC, the patient would be recommended to a multi-disciplinary team (MDT) to further assess the course of treatment with an intention-to-treat (ITT) assessment.

### Follow-up

Follow-up of all patients will be carried out according to our protocol (every 3 months for at least 2 years, every 6 months for years 3–5, then every 12 months for life). Physical examination, tumor marker examination, and abdominal CT are given at post-operative threshold and every 3 months. Endoscopic examination would be given every 1 year. Disease progression is defined as any direct signs (peritoneal thickening, nodular changes, mesenteric infiltration, pelvic masses etc.) or indirect signs (ascites, retro-peritoneal lesions, renal effusion or ureteral obstruction due to compression from recurrent masses) of local recurrence found on CT or signs of local recurrence during endoscopic examination.

#### Sample size calculation

**Fig. 2:**
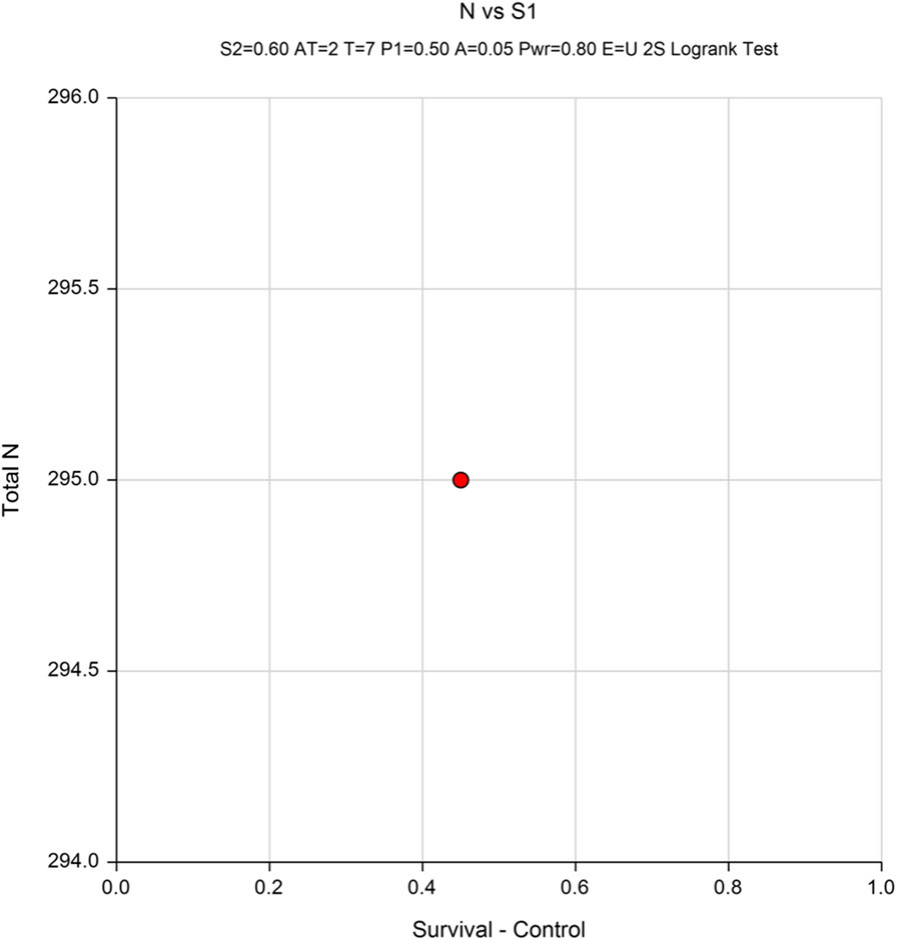
Logrank Test Power Analysis Chart Section

The main end point of this study is PFS. According to literature, the 5-year PFS of the operation control group is about 45% [26–28]; we speculate that with the L-HIPEC + NAC intervention, the 5-year PFS can be increased to 60% (estimating a 15% increase). The random distribution ratio of the experimental group and the control group is 1:1, the test level α is set to 0.05 on both sides, the statistical test efficiency (power) is set to 80% (β = 0.2). The expected study subjects will be enrolled for over 2 years and followed up for 5 years. Using PASS software version 11.0(NCSS, LLC. Kaysville, UT) for sample size estimation, the log rank test revealed a sample size of 295, which when factorizing a drop-out rate of 10%, was estimated to around 326, with 163 cases in each group (Fig. 2).

**Table:** 

Numeric Results in Terms of Events when the Test is Two-Sided and T0 is 5														
Power	Ctrl Evts E1	Trt Evts E2	Total Evts E	Haz Ratio (HR)	Ctrl Prop Surv (S1)	Trt Prop Surv (S2)	Accrual Pat’n	Accrual Time/Total Time	Ctrl Loss	Trt Loss	Ctrl to Trt	Trt to Ctrl	Alpha	Beta
0.8009	90.7	67.5	158.1	0.6397	0.4500	0.6000	Equal	2 / 7	0.0000	0.0000	0.0000	0.0000	0.0500	0.1991

### Statistical analysis

PFS is calculated from the date of randomization to the date of detected disease recurrence. Following events are defined as recurrence: primary cancer recurrence, newly diagnosed gastric cancer, and death. OS is calculated from the date of randomization to the date of death or date of last follow-up. Survival is estimated by using the Kaplan-Meier method, and differences between survival curves are examined with log-rank test. Fisher’s exact is used to compare patients’ characteristics between preoperative chemotherapy arm and preoperative.

## Discussion

This study is conducted to investigate the feasibility and efficacy of a more aggressive prophylaxis against PC in AGC patients undergoing gastrectomy of curative intent. This proposed study investigates a more comprehensive approach to the pre-surgical management of AGC where the inclusion of laparoscopic HIPEC is speculated to provide local management of occult peritoneal carcinomatosis or peritoneal free cancer cells and the use of NAC is speculated to induce tumor downsizing and down-staging. The high prevalence of peritoneal carcinomatosis after gastrectomies of curative intent in advanced stage patients has been prompting for more aggressive prophylactic treatment options to manage the spread of cancer cells due to lesion infiltration through the serosal or surgical trauma. The roles of intraoperative HIPEC and NAC have individually been investigated but the combination of laparoscopic HIPEC with NAC followed by surgery plus intraoperative HIPEC has not been investigated before and is an option worth exploring since it shows theoretical promise as a prophylaxis against PC after gastrectomy.

This is indeed the first prospective multicenter randomized study which will be investigating the comprehensive neoadjuvant role of HIPEC combined with NAC in the management of AGC patients at high risk for PC. The results of this study will contribute to establish treatment standards for clinical practice in AGC patients with and at risks of PC.

## Data Availability

The data and materials of the study will be made available on request.

## Abbreviations

AC: Adjuvant Chemotherapy
AGC: Advanced Gastric Cancer
ALT: Alanine aminotransferase
ASA: American Society of Anesthesiologists
AST: Aspartate aminotransferase
CTCAE: Common Terminology Criteria for Adverse Event
ECOG: Eastern Cooperative Oncology Group
GC: Gastric Cancer
Hb: Hemoglobin count
HIPEC: Hyperthermic Intraperitoneal Chemotherapy
IFCC: Intraperitoneal Free Cancer Cells
MDCT: Multiple detector computed tomography
MDT: Multi-disciplinary team
NAC: Neoadjuvant Chemotherapy
NYHA: New York Heart Association
OS: Overall Survival
PC: Peritoneal Carcinomatosis
PFS: Progression free survival
PLT: Platelet count
PTX: Paclitaxel
RCT: Randomized Controlled Trial
RECIST: Response Evaluation Criteria for Solid Tumors
SCr: Serum creatinine
SOX: S-1 and oxaliplatin
ULN: Upper limit of normal
WBC: White blood cells count
